# Influence of Antenatal Family Planning Counselling on Attitude Towards Early Postpartum Family Planning: A Randomised Controlled Trial in Kenya

**DOI:** 10.24248/eahrj.v9i2.858

**Published:** 2025-12-24

**Authors:** Morris Senghor Shisanya, Mary Kipmerewo, Everlyne Nyanchera Morema, Collins Ouma

**Affiliations:** a Kibabii University, Bungoma, Kenya; b Masinde Muliro University of Science and Technology, Kakamega, Kenya; c Maseno University, Maseno, Kenya

## Abstract

**Background:**

Globally, women value postpartum family planning (PPFP) for health and pregnancy spacing. It is imperative to understand the diverse aspects influencing women's attitudes towards early PPFP to address them effectively. There is a lack of comparative studies on the effectiveness of interventions to improve attitudes towards early PPFP. Bridging this gap is vital for evidence-based FP promotion and better maternal and child health outcomes. This study, therefore, compared attitudes toward early PPFP across nurse-led, community-based, and routine ANC groups.

**Methods:**

The study was a randomised control trial conducted in Kisumu County among pregnant women. Three arms were established: nurses’ and community interventions and a control. Sample size was determined based on expected differences in contraceptive use postpartum. Multistage sampling involving purposive, cluster, and simple random sampling was used. The intervention involved providing antenatal information on postpartum family planning (PPFP) using a mobile phone-based tool. Attitudes towards PPFP were measured using Likert scales and analysed through ANOVA. The study aimed to assess the impact of interventions on attitudes towards early PPFP.

**Results:**

Most participants (96.4%) had a positive attitude towards early PPFP, though some factors were linked to reduced positivity. Higher education (OR 0.6, 95% CI, 0.4 to 0.9, *P*=.026), comorbidity (OR 0.2, 95% CI, 0.1 to 0.6, *P*=.006), and longer counselling waiting and turnaround times (OR 0.9, 95% CI, 0.8–1.0, *P*=.059) and (OR: 0.9, 95% CI: 0.9 to 1.0, *P*=.032) were associated with more negative attitudes, while good perceived health increased positivity (OR 3.1, 95% CI, 1.0 to 9.2, *P*=.043). There was no significant difference in attitude between study arms (F (2,243) =3.0, *P*=.053).

**Conclusion:**

The study found a generally positive attitude towards early PPFP among participants, but no significant difference in attitude between intervention and control arms. Negative attitudes were associated with comorbidities, longer waiting times, and counselling turnaround times. The study recommends improvements in counselling quality by optimising waiting and turnaround times.

## BACKGROUND

Globally, many women recognise the importance of early postpartum family planning (PPFP) for spacing pregnancies and maintaining their health. Having a positive attitude towards contraceptive methods and FP practices soon after childbirth has an influence on FP interventions seeking and ultimate uptake. Two studies in India showed that while behaviour change interventions improved attitude and increased acceptance of early PPFP, prior FP use plays a critical role, as it shapes women's attitude towards early PPFP adoption.^1, 2^ This underscores the fact that while studying the influence of an intervention to change attitude towards early PPFP, it is important to contextualise the intervention's effects.

Contextualising FP interventions is supported by studies conducted in Africa that shed light on the multifaceted nature of women's attitudes toward early PPFP. Two such studies in diverse settings in Uganda and Ghana show that understanding the natural contraceptive effects of postpartum amenorrhoea interval and breastfeeding significantly influenced FP acceptance. Thus, understanding the contraceptive effects of breastfeeding and addressing barriers to accessing FP services play crucial roles in shaping women's attitudes toward early PPFP.^3^ Furthermore, positive partner attitudes toward FP correlated with increased FP service utilisation.^4, 5^ Equally, low knowledge of FP methods has been identified as a key factor contributing to low contraceptive utilisation in sub-Saharan African countries.^6^ Ethiopian studies indicated that knowledge, perception, and unmet needs for FP services significantly influenced women's attitudes and practices regarding PPFP.^6, 7^ One of the studies identified various factors, including women's age, pregnancy planning status, partner support, attitudes toward FP, and maternal satisfaction with care, as pivotal in immediate and early PPFP utilisation.^8^

In Kenya, the attitudes of women toward early PPFP exhibit a wide spectrum of perspectives. While some women recognise the importance of spacing pregnancies and using contraception during the postpartum period, challenges and barriers persist. These include inadequate postpartum care and a high unmet need for FP services, which hinder the development of positive attitudes towards FP, access and utilisation.^9, 10^ Moreover, missed opportunities for FP counselling during the antenatal period and cultural and societal factors significantly influence women's attitudes and their perceived need for contraception.^4, 11^ To address these challenges and improve attitudes toward early PPFP in Kenya, several strategies are essential. These include increasing community awareness about FP, promoting partner involvement in FP decisions, ensuring maternal satisfaction with antepartum and intrapartum care, and providing comprehensive and accurate information about the benefits of FP while addressing misconceptions.^8, 9^

The reviewed research on attitudes towards early PPFP shows a paucity of practical interventional studies that systematically compare the influence of antenatal interventions on women's attitudes towards early PPFP, particularly when it comes to community-based versus facility-based interventions. While existing research has provided valuable insights into factors affecting attitudes, there is a notable gap in understanding the differential impact of these two intervention approaches on shaping women's attitudes and subsequent behaviours regarding early PPFP. Addressing this research gap is essential for the development of evidence-based pragmatic strategies that can effectively promote FP during the postpartum period, ultimately contributing to improved maternal and child health outcomes. To address this gap, we conducted a three-arm randomised controlled trial (nurse-led, CHWled, and control) to compare how different antenatal PPFP information-giving approaches influence women's attitudes toward early PPFP. The primary objective of this study was to determine whether nurse-led or community-based antenatal PPFP information provision leads to a significantly different attitude toward early PPFP compared to routine antenatal care.

## METHODS

### Study Area and Design

This was a simple randomised control trial (RCT) with three arms carried out in facilities in Kisumu County among pregnant mothers in their second or third trimester attending ANC clinic in the study facilities or who are within the respective community units (CUs). The study was conducted in six centres. For the nurse-led and control arms, two primary health centres each, one rural and one urban—were used for enrolment, while the community arm recruited participants from two corresponding community units, one rural and one urban. The eligibility criteria for health centres included offering a continuum of ANC, delivery, and PNC services; provided at least three modern contraceptive methods; having no contraceptive stock-outs in the preceding six months; conducted a minimum of 10 deliveries monthly; and were willing to participate. Pregnant women eligible if they were in their second or third trimester, attended ANC with the intention of attending PNC, provided informed consent, and resided within 20 km of the health centre. Women were excluded if they were participating in another study, had latex sensitivity, were not expecting a male partner in the next 12 months, were unable to complete consent forms, or reported that their only male partner had undergone vasectomy.

### Sample Size Determination

The sample size was estimated using the following assumptions: among women three months postpartum, KDHS data reported 27% uptake of any contraceptive method (modern or traditional) in Kenya, while the CPR in the general population was 53%.^12^ These figures justified the assumption of a desired 26% difference between the control and intervention groups (i.e., a 26% increase in modern contraceptive uptake by three months postpartum). The sample size was therefore calculated pairwise for two separate comparisons: the community arm versus the control arm, and the nurse-led arm versus the control arm.^13, 14^ Rosner's (2015) formula for comparing differences in proportions, accounting for Type I and II errors and desired power, was applied. After adjusting for an anticipated 10% loss to follow-up, the final sample size was 246. This required recruiting 123 pregnant women in each setting (rural and urban), giving each of the six study facilities a target of 41 clients. Although the sample size was calculated based on uptake, it was also sufficient for attitude analysis.

### Sampling Technique

A multistage sampling approach was used. First, one sub-county with both rural and urban settings was purposively selected. Second, cluster sampling was used to select the intervention and control facilities, with facilities matched based on operational level. Third, eligible participants within each facility were selected through simple random sampling. Cluster random sampling was also applied to select the community units assigned to the intervention. Each eligible participant was then randomly assigned to the study using simple random sampling through a lottery method involving folded papers labelled “yes” or “no.” There was no blinding, as the intervention and control facilities were known.

### Intervention Procedure

The intervention involved providing antenatal information on postpartum family planning (PPFP) using a standardised, mobile-phone-based counselling tool. It was administered once, and the structured counselling guide ensured that all components of the WHO Medical Eligibility Criteria (MEC) for early PPFP methods were addressed. The tool, developed on Kobo Toolbox, offered a validated and structured guide that enabled the research team to monitor session duration and fidelity to the counselling procedure. The intervention was delivered during the second and third trimesters. Service providers at the facilities and in the community were trained on antenatal PPFP information provision prior to recruitment. Training was standardised using pre- and post-tests, and implementers were required to score at least 60% on the post-test. In all arms, study nurses conducted recruitment through history taking and physical assessment using the study's case report form (CRF). In the control arm, participants received routine ANC care. Although FANC recommends providing PPFP information during pregnancy, routine care typically offered such counselling in an unstructured, nonstandardised, and non-mandatory manner as part of general health education. No structured PPFP counselling was provided beyond usual practice. In the intervention arms, the study nurse or a Community Health Worker (CHW) used the phone-based counselling tool based on WHO MEC guidelines to provide PPFP counselling. Each session lasted at least 20 minutes. All clients completed an exit interview and were informed about the final follow-up interview scheduled for between 14 and 16 weeks postpartum. No additional concomitant care or intervention outside the prespecified criteria was recommended or prohibited during the trial. Participants were discontinued if they lost their only sexual partner, if the partner underwent vasectomy during pregnancy, or if the participant developed postpartum psychosis or was hospitalized for more than 14 weeks postpartum.

### Data Collection Procedure

**FIGURE 1:**
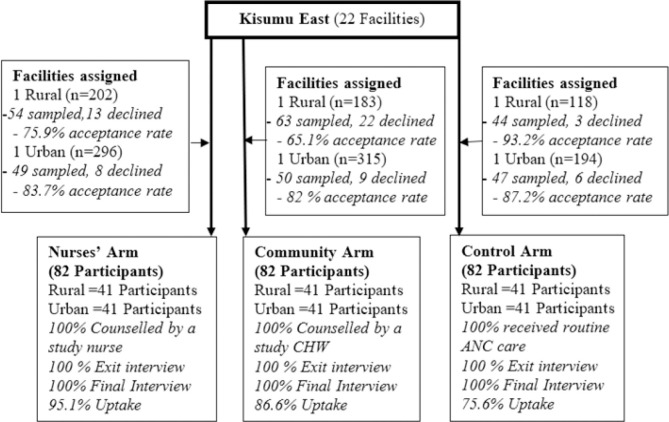
Consort Flowchart Showing Participant Flow Study Flow Diagram

The intervention phase of the study lasted from 26th February to 30th August 2022. Five tools were used for data collection, namely, the client exit interview guide, case report form, appointment card, site appraisal form and questionnaire. All the tools were used to collect quantitative data except the site appraisal form and some questions in the questionnaire that needed brief explanation. Figure 1 shows the flow of the study, including acceptance rates for those sampled. Each health centre and community unit had a trained research assistant who ensured adherence to the study manual and standard operating procedures for data management.

The primary outcomes were the intent to use early PPFP and actual uptake of FP methods at three months postpartum. Uptake of modern contraceptive methods was assessed between 14 and 16 weeks after birth. The CRFs were completed at recruitment by trained ANC providers. Appointment cards were completed by counsellors after clients agreed to a postnatal PPFP follow-up date. The questionnaire was administered at 14 weeks postpartum during the scheduled MCH visit, or soon thereafter, by a trained enumerator who was blinded to group assignment. A process evaluation was conducted to identify barriers and enablers related to PPFP delivery. This assessment drew on data from client exit interviews and site appraisal forms.

### Validity

The study ensured clarity in describing the population and outlines clear inclusion and exclusion criteria, while employing probability sampling to secure a representative sample, enhancing the generalizability of findings. Construct validity is strengthened by utilizing a questionnaire adapted from peer-reviewed research on family planning (FP) and incorporating questions from the Theory of Planned Behavior (TPB) official testing platforms. Content validity is assured by aligning data collection tools with a conceptual framework derived from the TPB, a well-established behavioral theory in FP research, effectively capturing intention-related aspects.

### Reliability

Interrater reliability was ensured by the researcher through training of data collectors. Internal consistency was assured by piloting the tools and refining them to ensure they captured the essence of what they were meant to collect, with acceptable Cronbach's alpha of 0.7.

### Analysis

Attitude toward early PPFP was directly measured using four 7-point Likert scale items (Cronbach's alpha 0.844), ranging from strongly agree (7) to strongly disagree (1). Attitude was then summarized into a continuous variable by calculating the mean of the Likert-scale item scores. The mean scores were categorized as follows: very negative attitude (≥1 to <2), moderate negative attitude (≥2 to <3), weak negative attitude (≥3 to <4), neutral attitude (≥4 to <5), weak positive attitude (≥5 to <6), moderate positive attitude (≥6 to <7), and very positive attitude (≥7). These were summarized using frequency tables and measures of central tendency and dispersion. Attitude was then included in an ordinal logistic regression analysis alongside participant characteristics and process indicators. To enhance interpretability and reflect the ordered patterns of FP utilization reported in the 2014 KDHS, certain sociodemographic variables were converted into ranked categories based on their nationally observed gradients, a decision intended to produce more reader-friendly regression outputs. Analysis of variance (ANOVA) of attitude toward PPFP, with post hoc tests for differences in means between study arms, was conducted. Tukey's post hoc test was used for variables that met the homogeneity of variance requirement (Levene's test p > .05). For variables that did not meet this requirement (Levene's test p < .05), the Brown–Forsythe adjusted ANOVA was reported, and Tamhane's post hoc test was used for pairwise comparisons. Partial eta^2^ was used to determine effect size.

### Ethical Considerations

The study was approved by the Masinde Muliro University of Science and Technology (MMUST) School of Graduate Studies (SGS) (Ref: MMU/COR:509099). Ethical clearance was obtained from the MMUST Institutional Ethics Review Committee (IERC) (MMUST/IERC/013/2021). Research authorization and a permit were acquired from NACOSTI (Ref. No. 522628). An official data collection permission letter was obtained from the County Director of Medical Services (DMS). The trial was registered with the Pan African Clinical Trial Registry (PACTR) under the number PACTR202109586388973, and the study protocols were published in PLOS ONE.^15^ Signed written informed consent was obtained from all participants after they were informed about the purpose of the study and their rights. To ensure confidentiality and privacy, participants’ names were not recorded in the CRFs, and data collection was conducted privately. The principles of justice and impartiality were upheld by ensuring equal opportunity for the target population to participate in the study through probability sampling. Additionally, refresher training was conducted for the nurses providing antenatal care services to clients in both the intervention and control arms, and guidelines were provided to ensure uniformity of the process.

## RESULTS

### Sociodemographic Characteristics of Participants

The mean age of the participants was 25.2 years (SD = 4.9), with a minimum of 16 years and a maximum of 42 years. The modal age group was 15 to 24 years. Most participants (84.1%) were married. The highest level of education attained was tertiary, and more than 63% of participants had completed high school or tertiary education. More than 86% of the participants earned less than 5,000 KES, and almost all were Christians.

### Attitude Towards Early PPFP among Study Participants

Individual attitudes towards PPFP were assessed using Likert scale indicators, and the overall rating was converted into a continuous variable by computing the mean score of the measurement parameters. These were summarized using frequency tables as well as measures of central tendency and dispersion. Overall attitude was categorized on the Likert scale as follows: very negative (≥1 to <2), moderate negative (≥2 to <3), weak negative (≥3 to <4), neutral (≥4 to <5), weak positive (≥5 to <6), moderate positive (≥6 to <7), and very positive (≥7). The average attitude rating towards early PPFP was high, at 5.8 (SD = 1.0).

**FIGURE 2:**
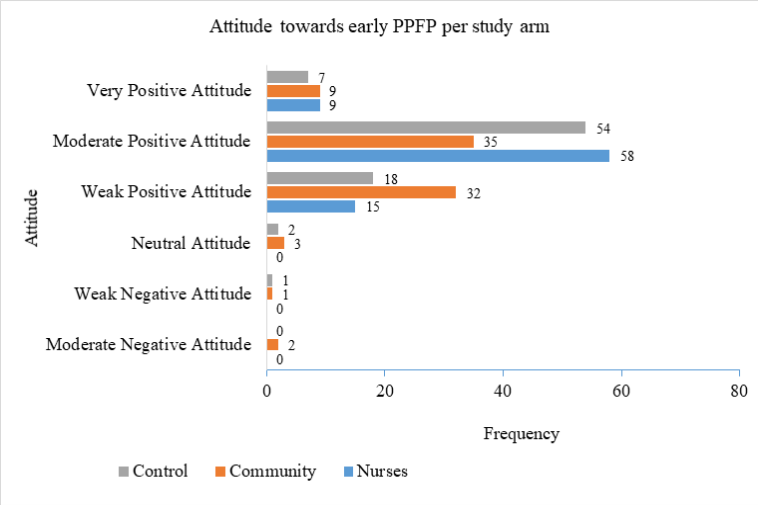
Attitude Towards Early PPFP Per Study Arm

Figure 2 shows that the average attitude was skewed towards positive, with 237 participants (96.4%) falling within the positive spectrum as follows: moderate positive attitude 147 (59.8%), weak positive attitude 65 (26.4%), and very positive attitude 25 (10.2%). The remaining 3.6% were distributed as follows: neutral attitude 2%, weak negative attitude 0.8%, and moderate negative attitude 0.8%.

### Predictors of Participants’ Attitude Towards Early PPFP

Table 1 shows a summary of the ordinal regression analysis of individual attitudes, with client-related aspects and counseling process aspects as predictors. Client-related aspects were further disaggregated into sociodemographic characteristics, pregnancy, labor and delivery aspects, previous FP experience, and intimate partner relationships. The 2014 KDHS demonstrated that FP utilization increased with age, level of education, and income quintiles, and that individuals who were employed or lived in urban areas had higher FP utilization rates compared to their counterparts. These KDHS trends informed the decision to convert level of education, marital status, and employment status into ranked variables. Groups with higher FP utilization, according to the 2014 KDHS, were assigned higher ranks; for example, as education level increased, FP utilization increased. Therefore, primary education was assigned a rank of 1, secondary education a rank of 2, and tertiary education a rank of 3. Counseling process indicators were mostly measured using a Likert scale.^12^ In the ordinal logistic regression model, each odds ratio reflects the change in the odds of being in a higher attitude category per oneunit increase in the respective predictor.

**TABLE 1:** Predictors of Participants’ Attitude Towards Early PPFP

Category	Parameter	OR	95% CI	*P Value*
Client related aspects	Clients Age	1.0	0.9 – 1.1	.109
	Level of education	0.6	0.4 – 0.9	.026
	Monthly income	1.3	0.7 – 2.3	.365
	Marital status	1.1	0.7 – 1.6	.753
	Employment status	1.3	0.9 – 1.9	.109
	ANC visits number	1.0	0.8 – 1.3	.798
	Gestation at recruitment	1.1	1.0 – 1.2	.099
	Comorbidity	0.2	0.1 – 0.6	.006
	Number of children	1.0	0.8 – 1.2	.920
	Health education in pregnancy	1.5	0.4 – 5.5	.485
	Complication during pregnancy	0.8	0.5 – 1.5	.536
	Labour complications	1.1	0.5 – 2.4	.824
	Postpartum complication	1.4	0.5 – 3.6	.511
	Health education afterbirth	1.2	0.6 – 2.4	.700
	Health status After pregnancy	3.1	1.0 – 9.2	.043
	Health status 3 months postpartum	0.6	0.2 – 1.7	.328
	Rating previous experience with FP	1.1	-8.6 – 1.1	.816
	Estimated cost of previous FP services	1.2	-8.5 – 1.3	.840
	Intimate partner relationship	1.1	0.9 – 1.4	.485
Process related aspects	FP counselling waiting time	0.9	0.8 – 1.0	.059
	FP Counselling turnaround	0.9	0.9 – 1.1	.032
	Mode counselled	1.1	0.6 – 1.9	.835
	Counselling Quality	1.0	0.9 – 1.1	.104
	Fidelity to process	0.9	0.6 – 1.3	.539
	Set postnatal appointment	1.2	0.6 – 2.3	.579

Ordinal regression analysis of predictors of participant attitude towards early PPFP, OR-Odds Ratio, 95% CI – 95% confidence interval, Significance set at *P*≤.05.

The sociodemographic aspects included in the ordinal regression model were participants’ age, marital status, level of education, employment status, and monthly income. Of these, only level of education had a significant effect on attitudes towards early PPFP uptake. As education level increased, the odds of having a positive attitude towards early PPFP decreased (OR 0.6, 95% CI, 0.4 to 0.9, *P*= .026). Age showed a similar but nonsignificant negative effect on attitude. Monthly income, marital status, and employment status had positive but non-significant effects on the odds of a positive attitude towards early PPFP as their assigned ranks increased.

Pregnancy-related aspects included in the ordinal regression analysis model were: number of ANC visits, gestational age at which PPFP was counselled, comorbidity, number of children, health education during pregnancy, and complications during pregnancy. Only comorbidity had a significant effect on attitude towards early PPFP uptake (OR 0.2, 95% CI, 0.1 to 0.6, *P* =.006).

Labour, delivery, and postpartum-related aspects included in the ordinal logistic regression model were: health education after birth, labour complications, postpartum complications, and health status at three months postpartum. Perceived health status after pregnancy was the only significant determinant of attitude towards early PPFP. Participants who rated their health as good were more than three times as likely to have a more positive attitude towards early PPFP compared to those who rated their post-pregnancy health as poor (OR 3.1, 95% CI 1.0 to 9.2, *P*= .043). Labour complications, postpartum complications, and health education after birth also had a positive effect on attitude, although these were not statistically significant.

Ordinal logistic regression analysis of previous experience with FP and the estimated cost of previous FP services did not show any significant effect on attitude towards early PPFP utilization. However, both factors had a positive influence on attitude towards PPFP. Although there was a positive relationship between intimate partner relationship and attitude towards early PPFP, the effect was not statistically significant (OR 1.1, 95% CI 0.9 to 1.4, *P* = .485).

Process-related aspects included in the ordinal regression model were: FP counselling waiting time, counselling turnaround time, mode of counselling, counselling quality, and fidelity to process. FP counselling waiting time had a borderline effect, while counselling turnaround time had a significant effect on attitude towards early PPFP. As both counselling waiting time and turnaround time increased, the odds of a positive attitude towards PPFP decreased (OR 0.9, 95% CI, 0.8 to 1.0, *P* = .059; and OR 0.9, 95% CI, 0.9 to 1.0, *P* = .032, respectively).

### Effect of Intervention on Participants’ Attitude Towards Early PPFP

Table 2 shows the differences in the distribution of attitudes towards early PPFP by study arm, which were assessed using ANOVA. A summary of descriptive statistics for each arm was also conducted as a preamble to the main ANOVA test. Partial eta-squared (η2\eta^2η2) was used to estimate the between-arm effect size for ANOVA.

**TABLE 2:** ANOVA of Attitude Towards Early PPFP Between Study Arms

Study Arm		MD	95% CI	*P Value*	Effect Size
A	B	(A-B)			
Intervention	Control	-0.2	-0.5 – 0.10	.106	0.01
Nurses’	Community	0.3	-0.1 – 0.7	0.167	0.02
Nurses’	Control	-0.1	-0.5 – 0.3	0.868	0.00
Community	Control	-0.4	-0.7 – 0.1	0.055	0.03

A and B are column labels, MD-Mean difference between A and B; 95% CI is the Confidence Interval for the Mean Difference (MD); Effect size was estimated by Partial eta^2^ (0.01 to <0.06 - Small, 0.06 to <0.14 medium, ≥0.14 Large); Tukey's post hoc test was applied because homogeneity of variance was met, Significance set at *P*≤.05

The nurses’ arm had a mean attitude score of 5.9 (SD = 0.9), the community arm had a mean score of 5.6 (SD = 1.1), and the control arm had a mean score of 5.9 (SD =1.0). Across all study arms, the total mean attitude score towards PPFP was 5.8 (SD = 1.0).

One-way ANOVA for attitude towards PPFP was done with Levene's test showing that homogeneity of variance was met F (2,243) =1.0, *P*=.380. Therefore Tukey's post hoc test would be used to estimate significance of mean difference, effect size between arms. The ANOVA for attitude towards PPFP and study arms revealed no statistically significant difference in the mean attitude scores between arms F (2,243) =3.0, *P*=.053.

## DISCUSSION

Attitudes toward early PPFP were already overwhelmingly positive in this population, with 96.4% of participants expressing positive attitudes, which helps explain why the interventions did not produce additional changes; this suggests that attitude may not be the primary barrier to early PPFP uptake, shifting attention instead to factors such as access, cost, and service quality. Other studies showed comparable findings, with studies in Ethiopia and Bangladesh showing that 90% and 78% of the participants expressed a positive attitude and interest in using an FP method after delivery, respectively.^16, 17^ A Nigerian study found that a majority of the participants had a positive attitude towards PPFP, with the majority indicating that they would prefer to receive FP services immediately after childbirth.^18^

While these studies showed the level of attitude towards early PPFP, they did not show what factors influenced attitude. Attitude towards PPFP can be influenced by a wide range of factors; for this study, factors that could potentially influence attitude were clustered into sociodemographic factors, obstetric history, past experience with FP, intimate partner relationship, and process-related aspects. The current study shows that as the level of education increased, the odds of having a positive attitude towards early PPFP decreased. This suggests that individuals with higher levels of education may be less likely to have positive attitudes towards early PPFP. It is possible that there may be other factors that are correlated with both education level and attitude towards early PPFP, and that these factors may be responsible for the observed relationship between the two variables. As much as this is an unusual finding that requires further investigation and may be context-specific, it is noteworthy that individuals with higher levels of education may be more likely to have access to misleading information and myths about PPFP and their side effects or cost, which may influence their attitudes towards early PPFP, as was shown in a study by Sedgh & Hussain (2014), which hinted at potential misconceptions accompanying higher levels of education.^19^ Therefore, it is important to consider these and other potential confounding factors when interpreting the results. Bekele and others (2020) and Elweshahi and others (2018) had contrary conclusions where higher levels of education positively influence attitude towards use of postpartum FP and reduced unmet need for FP.^20, 21^

Participants with comorbidities had a significantly more negative attitude towards early PPFP uptake compared to those without comorbidities. Furthermore, individuals who rated their health as good were more than three times as likely to have a positive attitude towards early PPFP than those who rated their post-pregnancy health as poor. Individuals with comorbidities may be more focused on managing existing medical conditions and may have less time or energy to devote to learning about and considering family planning (FP) options. Additionally, they may face financial or logistical barriers to accessing FP resources, which could impact their attitudes towards early PPFP.^22^ Therefore, it is important to consider these and other potential factors when interpreting study results and designing interventions to promote women's attitudes towards early PPFP services.^23^ On the other hand, a study among HIV-positive patients with other comorbidities, such as sexually transmitted diseases, found a positive association with attitudes towards FP and actual FP use.^24^

Although a good intimate partner relationship was not shown to have a significant positive effect on attitudes towards early PPFP in our study, other research has indicated that individuals in supportive partner relationships are more likely to discuss and make joint decisions about FP, often resulting in more positive attitudes towards early PPFP.^25^ Additionally, individuals with strong partner support may feel more empowered to make decisions about their own health and well-being, which can further influence their attitudes towards early PPFP.^26^ Our findings therefore suggest that, in this population, partner relationships may not be a primary driver of PPFP attitudes, highlighting the need for further research using more sensitive or context-specific measures.

Longer waiting and turnaround times for counselling were associated with more negative attitudes towards early PPFP. This may be due to factors such as frustration, inconvenience, or the perception of being rushed through the counselling process. On the other hand, shorter waiting and turnaround times for counselling may be associated with more positive attitudes towards early PPFP, as they are perceived as more efficient and convenient.^27, 28^ It is therefore imperative that targeted interventions are carefully designed to address the specific needs and challenges faced by individuals seeking counselling, particularly regarding optimizing counselling turnaround time and reducing waiting periods.

Analysis of differences in attitudes towards PPFP between study arms revealed no significant difference in mean attitude scores, echoing Rossier and others (2014), who acknowledge the complex nature of attitudes toward FP. However, a cross-sectional study in Ethiopia showed that a positive attitude increased the odds of FP uptake among women of reproductive age (WRA).^21^ Although this was an observational study conducted in a general population of WRA, its findings highlight the importance of attitude in influencing early PPFP uptake. The findings by Bekele and other (2020) are supported by other observational studies in different settings.^29, 30^ While these studies do not compare the effectiveness of antenatal FP interventions in aligning women's FP attitudes for better early PPFP uptake, they underscore the complexity of understanding women's attitudes toward FP. This calls for further research aimed at comprehensively understanding attitudes to inform multifaceted strategies for promoting early PPFP.

This similarity in effect between the nurses’ arm and the community arm is likely explained by the overwhelmingly positive baseline attitudes observed across the study population, which created a ceiling effect that limited the potential for either intervention to produce measurable change. These findings indicate that, in this context, improving early PPFP uptake may require strategies that address factors beyond attitude, such as service access, counselling quality, and health system barriers, rather than focusing solely on modifying women's attitudes.

### Study Limitations

It is important to note that the findings of this study should be interpreted within the context of its limitations, including the study setting and the generalisability of the results to other populations. The study is subject to selection bias, as it focuses exclusively on individuals attending antenatal care (ANC) clinics, which may limit the applicability of the findings to the broader pregnant population. A more diverse sample beyond ANC attendees would enhance external validity. Additionally, the study is limited by its recruitment of participants primarily in the third trimester, preventing assessment of the intervention's impact earlier in pregnancy and missing insights into factors influencing family planning decisions in the early stages.

## CONCLUSIONS AND RECOMMENDATIONS

There was a highly positive attitude toward early PPFP among the study participants. However, the study found no difference in attitude between the intervention and control groups. Having a comorbidity, longer waiting times, and extended turnaround times for counselling were associated with more negative attitudes toward early PPFP.

The study highlights the need for targeted information campaigns to address negative attitudes associated with comorbidities, emphasising the safety and benefits of early PPFP for individuals with comorbid conditions. Additionally, the healthcare system should implement measures to improve the quality of counseling by reducing waiting times and optimising turnaround times, thereby enhancing overall attitudes toward early PPFP.
